# Collodion in Erysipelas

**Published:** 1854-05

**Authors:** Hiram Nance

**Affiliations:** Lafayette, Illinois


*Collodion in Erysipelas.* By Hiram Nance, M. D., of Lafayette, Illinois. Read before the Stark County Medical Society.

I am happy to add for the good of mankind and the success of our profession, my experience (though limited,) in the use of Collodion in Erysipelas. All Physicians who are familiar with this disease look on it with suspicion, though trifling it may appear at its commencement or formative stage. Erysipelas appears in so many forms, and such variety of grades, that I believe few Physicians or Surgeons would attempt in a short essay like the present to give the different varieties. The kind that I refer to I will explain by citing a few cases.

*Case* 1st. I was summoned on the 11th day of Dec., 1852 to see J. M——, aged 16 years. He was of a nervo-lymphatic temperament; I found him with his upper lip much swollen, and on making inquiries his mother informed me that there always existed a fisure in the part that the disease seemed to commence at, and that he was quite subject to sore lip. The lip was swollen so much that it was entirely out of its ordinary shape, the focus of disease seeming to be the fisure above mentioned. Connected with the swelling there seemed to be a point containing matter near the center of the disease; this point was covered with a whitish eschar, but on puncturing it with a lancet but a very small quantity of matter would exude. After two or three days, as the swelling increased from the lip up to the nose, cheek, and on the scalp over the parietal bone, these little whitish points increased in number, but varied nothing in description. The swelling was enormous on that side of the face and head; so much so that his physiognomy was entirely changed, and he would not have been recognized by his nearest friends. The tumefaction also extended into his mouth, being so great that it was with great difficulty that the act of deglution could be performed.

The type of sympathetic fever was neither sthenic nor asthenic, being of a medium grade—pulse about 100—profuse perspiration most of the time, but little delirium at first; but as the disease advanced he became quite wild and incoherent, no intermission of fever, but slight remission—Bowels torpid.

*Treatment.*—Gave 8 or 10 grs. Prot. Chlo. Hydr., to be followed in 4 hours, with Sul. Magnesia ℥ss. every 2 hours until the Bowels are moved. After this I gave Sol. Tar. Emet. and a Dover Powder to quiet the system. Cold applications were applied to the head, and cold lead water applied in saturated cloths to the diseased parts. This local application I applied until I was satisfied that it produced no favorable effect. I then used Nit. Sil. in substance, and a strong solution until I was confident that nothing important could be gained from it. Poultices &c., were applied cold and hot, but all to no use.

I omitted to say that I gave Quinia at the intermission, but it shared the same fate with the other remedies, being destitute of any perceptible value. This treatment was continued until the fifth day and my patient grew more and more delirious with contracted pupils, and expired with a diseased brain, connected with the Erysipelas. I did not see this patient until he had been ill some three or four days.

*Case* 2.—About ten days after this time, E. H——, a Gentleman of about 40, was attacked in the same way. He had been out all one night and was exposed to a North West wind; on the third day he was taken with chilly sensations, succeeded by fever, and the lip commenced swelling; on the second day after this I was called, and I resolved to try something new, and as Collodion had been mentioned in the Journals, I determined to give it a trial. I made an application of it all over the swelling—gave him the same constitutional treatment I did the other—ordered the Collodion to be applied every four or five hours. At my next visit on the succeeding day I was agreeably surprised to find patient better. There had been no increase in swelling, the pulse was slower, not much head ache. I ordered Quinia and kept the Bowels moved by Pulv. Rhei and continued the Collodion.

Third day, patient still better, but complained of tension of the skin, where the Collodion was applied, and wished a different application, and I was induced by his solicitations to make a different application, and dressed the parts with Unguentum Hydr. Fortis, with constitutional treatment the same, with an addition of wine.

Fourth day, patient not so well, swelling much increased, some more fever; removed the ointment and again applied the Collodion; constitutional treatment continued.

Fifth day, improvement again, and so continued until perfect health was established.

*Case* 3.—A week after this time a Brother of the above was taken with a chill, succeeded by a fever; and the upper lip commenced swelling. I was called immediately, and ordered Collodion to be applied every four hours; same constitutional treatment as the others. The swelling never increased any more, and of course convalescence was established immediately.

*Case* 4.—I was called in the month of April, 1853, to visit Mrs. R——, and found her with her face much swollen—had had Erysipelas before; fever asthenic; ordered a laxative of Calomel and Rhei, followed by Quinia and wine, and $\frac{1}{4}$ gr. Sul. Morph. every three or four hours during the night, to quiet the system and promote sleep; with Collodion applied all over the face, to be repeated every three or four hours. I visited her three days, and convalesence was fully established.

*Case* 5.—Six days from this time I was called to see J. R. R. husband of the lady last reported. The disease had been progressing for four days; the middle finger of the left hand was the seat of the mischief; sthenic grade of fever; ordered Calomel Jallap and Cream Tartar—Collodion applied all over the hand, the swelling and distension being so great, I sacrificed with some ease to my patient the most painful part. When the fever remitted I gave Quinia. Under this treatment my patient improved until health was established.

---

April Meeting. Gentlemen.—Since I last had the pleasure of meeting with you, in our County Medical Society, I have had an opportunity to test farther the virtues of Collodion in Erysipelas.

On the 26th day of February 1854 I was called to see Wm. S——, forty years of age; found him suffering from zona (shingles). The eruption was in groups, from six or eight inches, to not more than an inch square in surface, spreading from the right hypochondriac region, to as far up as on the neck. I ordered a mild cathartic, to be followed by a dover powder, and if this did not quiet the system, to give morphia every three or four hours. As a local application I used a solution of Nitrat. Argent.; this remedy I have frequently used before in this disease, but the disease under its use will continue several days. I used it for three days with but little, if any benefit in this case. My patient became discouraged as well as myself, and I determined to try Collodion; accordingly I made a free application all over the eruption and ordered it to be applied twice a day. In 24 hours I was agreeably surprised to find my patient sitting up, saying that the "burning" had entirely ceased. I told him to continue it a few days longer; he did so, and the cure was perfect.

It is true that zona is not genuine Erysipelas, but should the remedy in subsequent cases prove so valuable it may save much suffering. Zona is a disease of very inferior importance, when compared with Erysipelas.

A child aged six years, of a scrofulous disposition, with the *Glandula Concatanata* of the lower jaw, in a state of supuration, was brought to me for treatment. I placed it on solution of Ferri Iodini, and the Tinct. of Iodine, to be painted on the parts that were not in a state of supuration. Five days from this time the child was presented again with very marked improvement in the glandular disease; but a herpetic eruption not unlike zona had made its appearance on the arm and forearm. I continued the Iodine treatment, and used Collodion, locally; improvement commenced immediately. It may be premature in me to report these two last cases, but should any member have similar ones, it would be a pleasure to me to have them test the virtues of Collodion and give me their experience.

On the 15th of march, 1854, I was called to see Mrs. H——, who was suffering under Erysipelas of the upper lip; fever, asthenic. I ordered mild cathartic of Calomel and Rhei. to be followed during the remission of fever, with Sul. Quinia in 2 gr. doses, every two hours, with local application of Collodion all over the lip, cheek, and up on the temples as far as the tumefaction extended. After the Collodion was applied, the swelling increased but very little.

Gentlemen, you will observe the first case that came under my care died. It is true that the disease had existed longer than any one, except one, before I saw him. Collodion was not used in his case. Had it been used, I believe my patient would now be in the enjoyment of good health.

You will also observe that I was induced in the second case to omit the Collodion, and on my next visit my patient was much worse; that I again prescribed it, and a continual improvement took place until convalesence was perfectly established.

In conclusion, I would say *use it* and *use* it *freely*, and I believe you will find a remedy that you will all confide in.

The makers, venders, &c. of Collodion recommended it for burns, scalds, fissures &c.; also as an adhesive agent. I would remark that I believe it not fit for any of these diseases, as the pain it causes in these, preponderates over all good that we derive from it.

*Query.* How does Collodion act upon the system or the diseased parts, to modify the disease so remarkably?

In answer to this I would say that I believe its favorable action is produced by an entire exclusion of all irritants acting immediately upon the diseased parts.

In most works or essays written upon Erysipelas you find various local applications recommended, such as Unguent. Hydr. Fortis, Adeps or Lard, Flour, Starch, Blisters, Nitrate of Argent. in substance or strong solution &c. &c., none of which so effectually excludes all irritants as Collodion. Of all the irritants that act upon Erysipelas none are probably so pernicious as the atmospheric air. Some authors have remarked the application of Lard was probably as valuable as any local agent, and I believe until Collodion was used it had equal advantages with any.

I don't wish to lay to much stress upon this valuable therapeutical agent, and place our whole and only hope upon it alone. No, Gentlemen, look to the system, and treat it upon a sound pathology. If there is a miasmatic state, then in conjunction with the local application of our favorite remedy, administer Calomel, Quinia &c. If you find a highly phlogistic state of the system, open a vein and reduce the person to the state you wish. If there is asthenia in an anæmic patient, administer wine, steel, &c.

